# Functional Characterization of a Novel *PBX1* De Novo Missense Variant Identified in a Pediatric Patient with CAKUT

**DOI:** 10.3390/genes16111346

**Published:** 2025-11-07

**Authors:** Caterina Scolari, Angelo Corso Faini, Giulia Verra, Martina Migliorero, Giulia Margherita Brach Del Prever, Claudia Saglia, Fiorenza Mioli, Carmelo Maria Romeo, Tullia Carradori, Maria Luca, Francesca Arruga, Francesca Mattozzi, Licia Peruzzi, Silvia Deaglio, Tiziana Vaisitti

**Affiliations:** 1 Department of Medical Sciences, University of Torino, Via Santena 19, 10126 Turin, Italy; caterina.scolari@unito.it (C.S.); angelocorso.faini@unito.it (A.C.F.); giulia.verra295@edu.unito.it (G.V.); martina.migliorero@unito.it (M.M.); giuliamargherita.brachdelprever@unito.it (G.M.B.D.P.); claudia.saglia@unito.it (C.S.); fiorenza.mioli@unito.it (F.M.); carmelomaria.romeo@unito.it (C.M.R.); tullia.carradori@unito.it (T.C.); maria.luca@unito.it (M.L.); tiziana.vaisitti@unito.it (T.V.); 2 Immunogenetics and Transplant Biology Service, Città della Salute e della Scienza Hospital, 10126 Turin, Italy; francesca.arruga@unito.it; 3 Pediatric Nephrology, Regina Margherita Children’s Hospital, Department of Public Health and Pediatric Sciences, University of Torino, 10126 Turin, Italy; fmattozzi@cittadellasalute.to.it (F.M.); licia.peruzzi@unito.it (L.P.)

**Keywords:** congenital anomalies of the kidney and urinary tract, genetic variant, functional validation

## Abstract

Background: Genetic variants in *Pre-B cell Leukemia Factor 1 (PBX1)* transcription factor (TF) have been associated with Congenital Anomalies of the Kidney and Urinary Tract (CAKUT). This study aims to functionally characterize a novel missense variant in a 4-year-old patient presenting with horseshoe kidney with preserved function, in the absence of a positive familial history. Methods: Clinical exome sequencing was performed on a 4-year-old child, followed by Sanger sequencing and family segregation studies to validate the identified variant. Functional assays to study the protein expression, molecular interactions and localization were then performed. Results: Genetic analysis identified a novel de novo variant [c.712C>T, p.(Arg238Trp), NM_002585.3], mapping in the first nuclear localization signal (NLS) of PBX1. When introduced in HEK293T cells, *PBX1*^c.712C>T^ did not affect protein expression, which was comparable to the wild-type (WT) counterpart. Similar results were obtained when modeling a missense variant [c.863G>A; p.(Arg288Gln)], located in the second NLS of the protein, previously reported in the literature but never functionally characterized. As a TF, PBX1 may work in association with MEIS and PKNOX1/2 cofactors, but none of the two variants modified the interactions with its cofactor PKNOX1. However, both variants significantly affected the nuclear localization of PBX1, increasing its retention in the cytoplasm while limiting its availability in the nucleus. Conclusions: In conclusion, we identified a novel de novo heterozygous missense variant in *PBX1* that impairs nuclear localization of the protein, potentially limiting its role as a TF and possibly explaining the clinical phenotype of the patient.

## 1. Introduction

Congenital anomalies of kidney and urinary tract (CAKUT) are a heterogeneous group of syndromes characterized by abnormalities affecting the kidneys or other structures of the urinary tract, potentially leading to kidney failure [1,2]. The clinical presentation ranges from severe malformations, including renal agenesis with or without hearing loss, abnormal ears, or developmental delay, to milder manifestations. CAKUT account for up to 30% of antenatally diagnosed congenital abnormalities [3,4] and at least 50% of cases of chronic kidney disease (CKD) that manifest within the first three decades of life [5]. Increasing evidence indicates that a significant proportion of CAKUT are genetic in origin. The genetic landscape of CAKUT has evolved in recent years, with more than 50 genes identified as monogenic causes of CAKUT, contributing to 12–20% of the etiology of the disease [6,7,8]. Additionally, pathogenic copy number variants (CNVs) have also been reported as disease mechanisms in CAKUT in 4–11% of patients [9,10,11]. More recently, epigenetic and environmental interactions were shown to contribute to interindividual heterogeneity, expanding the complexity of the disease [4,12]. Epigenetic regulation represents an intriguing pathogenetic mechanism as it tunes gene expression without altering the DNA sequence. Given that a significant number of genes that play a role during nephrogenesis are transcription factors (TFs), regulation of gene expression through epigenetic modifications may contribute to the heterogeneous etiology of CAKUT [13,14].

*Pre-B cell leukemia transcription factor 1 (PBX1)* gene is among the TFs associated with CAKUT [2]. It codes for a three-amino acid loop extension (TALE) protein that regulates gene expression during organ development. Because of its widespread expression, individuals with CAKUT resulting from *PBX1* variants often exhibit extrarenal manifestations [15,16,17,18]. PBX1 acts through the formation of nuclear complexes with other TALE and/or homeobox proteins [19]. Through its protein–protein interacting domain, PBX1 binds to MEINOX members, including Myeloid Ecotropic Integration Site (MEIS) 1–3 and PBX-regulating protein 1 (PKNOX1). In addition, PBX1 recruits chromatin accessibility regulators, finely tuning its activity as activator or repressor of transcription of its target genes [20,21]. PBX1 is strictly required for embryonic development as its full knockout results in non-viable embryos, underlying its central role as a key developmental regulator [19,22]. On the other hand, heterozygous germline variants in the gene result in complex and heterogenous phenotypes. Specifically, loss-of-function variants (frameshift, nonsense, partial or whole gene deletions), accounting for approximately 70% of PBX1 genetic alterations, have been associated with neurodevelopmental disorders, central nervous system anomalies, cardiac/renal and urinary malformations and skeletal anomalies. On the other side, missense variants are correlated to milder renal and urinary tract phenotypes and to less prevalent central nervous system malformations. Genetic variants map in different critical domains of PBX1, including the protein-binding domain or the homeodomain, compromising their ability to form heterodimers and to bind to DNA, respectively. It has also been shown that variants mapping in the nuclear localization (NLS) or nuclear export signal (NES) modulate its cytoplasmic retention, contributing to CAKUT, intellectual disabilities, congenital heart diseases and multiple organ malformations phenotypes [19].

This work reports the identification and functional characterization of a novel missense variant in the *PBX1* gene, diagnosed in a 4-year-old patient presenting with a mild syndromic condition. Results provide evidence of its pathogenetic role, expanding the list of *PBX1* causative variants.

## 2. Materials and Methods

### 2.1. Clinical Exome Sequencing

Clinical exome sequencing (CES) was performed as previously described [23,24]. Briefly, libraries were prepared using the TruSight One Expanded Sequencing Kit enriched for the coding regions of 6700 genes with clinical relevance (Illumina, San Diego, CA, USA) following manufacturer’s instructions. DRAGEN software v4.3 (Illumina, San Diego, CA, USA) was used for reads alignment, single nucleotide, CNV and structural variants calling. Variant prioritization was performed with the Geneyx software v5 (Geneyx, Herzliya, Israel).

### 2.2. Sanger Sequencing

Validation of the CES identified variant (c.712C>T, R238W, NM_002585.3) and family segregation study were performed by Sanger sequencing. Primers were designed via the Pick Primers TOOL of NCBI (https://www.ncbi.nlm.nih.gov/tools/primer-blast/; access on 14 February 2022). The following primers were used: FW 5′-TAGCGTTGGTTTTGGCATCC-3′, REV 5′-TGCTTACCTGGGAGACTGTG-3′ (Eurofins MWG Operon, Ebersberg, Germany).

### 2.3. Literature Review

A literature review and genetic databases search was performed to obtain a list of known *PBX1* genetic variants. Specifically, we interrogated the following main genetic variants databases: ClinVar (https://www.ncbi.nlm.nih.gov/clinvar/; accessed on 13 December 2024), Franklin by Genoox (https://franklin.genoox.com; accessed on 13 December 2024), Varsome (https://varsome.com; accessed on 13 December 2024) and publications in the literature using the Mastermind Genomic Intelligence Platform database (https://mastermind.genomenon.com; accessed on 13 December 2024). Identified variants were filtered using the American College of Medical Genetics and Genomics (ACMG) classification criteria and including C3, C4 and C5 variants.

### 2.4. Site-Directed Mutagenesis and Sequence Analysis

The (c.712C>T, R238W) and (c.863G>A, R288Q) genetic variants were introduced into a turbo GFP-tagged plasmid encoding the wild-type *PBX1* gene (NM_002585.4; Human Tagged ORF Clone, Origene, Rockville, MD, USA) using the QuikChange Lightning Site-Directed Mutagenesis Kit (Agilent, Santa Clara, CA, USA). The following primers were used: FWD: 5′-CGGCGGAAGAGATGGAATTTCAACA-3′; REV: 5′-TGTTGAAATTCCATCTCTTCCGCCG-3′ for the *PBX1* R238W variant and FWD 5′- TTGGAAATAAGCAAATCCGGTACAA -3′; REV: 5′-TTGTACCGGATTTGCTTATTTCCAA-3′ for the *PBX1* R288Q variant. The *PBX1^R238W^* or *PBX1^R288Q^* clones were verified by Sanger sequencing (Eurofins Genomics, Milan, Italy), following DNA extraction using the QIAprep Spin Miniprep Kit (#27106 Qiagen, Milan, Italy) following manufacturer’s instructions. The following primers were used: FWD: 5′-GCATCATCCACCGCAAGTTC-3′; REV: 5′-TTGAGGGCGAGTTAGCTTGG-3′ for the *PBX1* R238W variant and FWD 5′-CCGATTTCTGGATGCGCGG-3′; REV: 5′-TTGAGGGCGAGTTAGCTTGG-3′ for the *PBX1* R288Q variant.

### 2.5. Cell Culture and Transient Transfection

HEK293T cells were cultured in RPMI-1640 medium, with L-glutamine and sodium bicarbonate, (Sigma-Aldrich, Milan, Italy) supplemented with 10% (*v*/*v*) fetal bovine serum (ThermoFisher Scientific, Milan, Italy) and incubated in a humidified 5% CO_2_ incubator at 37 °C. Clones were routinely tested for mycoplasma contamination. To model the (c.712C>T, R238W) and (c.863G>A, R288Q) variants, HEK293T cells were transiently transfected with either wild-type (WT)-*PBX1* or R238W or R288Q mutant *PBX1* with Lipofectamine™ 3000 Transfection Reagent (ThermoFisher Scientific, Milan, Italy). Transfection efficiency was assayed by flow cytometry analysis checking GFP expression (FACS Celesta, BD Biosciences, Milan, Italy).

### 2.6. Western Blot and Immunoprecipitation Antibodies

Antibodies used for Western Blot were anti-PBX1 (#4342, Cell Signaling Technology, Beverly, MA, USA), anti-MEIS1/2 (#12744S, Cell Signaling Technology, Beverly, MA, USA), anti-PKNOX1 (NBP2-19853, Novus Biologicals LLC, Milan, Italy), anti-PKNOX2 (#H00063876-M01, Abnova Corporation, DBA, Milan, Italy), anti-Actin (#sc-47778, Santa Cruz Biotechnologies Inc., Dallas, TX, USA), anti-Lamin A/C (#4777S, Cell Signaling), anti-α-Tubulin (#3873S, Cell Signaling), anti-phosphoCREB Ser133 (#9198, Cell Signaling Technology, Beverly, MA, USA), followed by an anti-mouse (#NA931V, Cytiva; Euroclone, Milan, Italy) or anti-rabbit (#NA94V, Cytiva, Euroclone, Milan, Italy) HRP-conjugated secondary antibody. The antibody used for immunoprecipitation was the anti-PBX1 (PCRP-PBX1-3C8; #A278320, Antibodies.com, Sial, Rome, Italy). Images were acquired using ChemiDoc Imaging System (BioRad, Milan, Italy).

### 2.7. Total Lysates, Nuclear-Cytoplasm Fraction and Western Blot Analyses

To evaluate PBX1 expression, WT, PBX1^R238W^ or PBX1^R288Q^ HEK293T cells (1 × 10^6^) were lysed, as previously reported [25], followed by Western blot analyses. To analyze PBX1 localization, cytoplasmic and nuclear fractions from WT, PBX1^R238W^ or PBX1^R288Q^ HEK293T cells (1 × 10^6^) were obtained using the NE-PER™ Nuclear and Cytoplasmic Extraction kit (ThermoFisher Scientific, Milan, Italy). Western blot analysis was then performed either on total or fractionated cell extracts using the 10% Mini-PROTEAN^®^ TGX™ Precast protein gel (BioRad, Milan, Italy), followed by blotting into nitrocellulose membrane (BioRad). Protein bands were quantified using Image Lab Software v6.01 (BioRad, Milan, Italy). Nuclear fractions were normalized on Lamin A/C and the ratio between mutant and WT counterpart was calculated. Where indicated, WT, *PBX1^R238W^* or *PBX1^R288Q^* HEK293T cells were treated with 60 microM forskolin (FSK; Sigma-Aldrich, Milan, Italy) for 2h before further analyses.

### 2.8. Immunoprecipitation

Total cell lysates (300 µg) were precleared with DynaGreen Protein A magnetic beads (30 min, 4 °C, #80102G, ThermoFisher Scientific; Milan, Italy) and incubated with anti-PBX1-coated beads (90 min, 4 °C). Immunocomplexes were then extensively washed with a PBS-Tween 0.1% buffer, eluted with 50 mM glycine pH 2.9 and analyzed by Western blot.

### 2.9. Confocal Microscopy

WT, *PBX1^R238W^* or *PBX1^R288Q^* HEK293T cells (1 × 10^5^) grown on coverslips were fixed in 4% paraformaldehyde (Sigma-Aldrich, Milan, Italy) for 15 min and permeabilized with 0.25% Triton X-100 (Sigma-Aldrich, Milan, Italy) for 20 min, both at room temperature. Cells were stained with 4,6-diamidino-2-phenylindole (DAPI; #D3571, ThermoFisher Scientific, Milan, Italy) and mounted on a slide using a fluorescence mounting medium (ProLong, ThermoFisher Scientific, Milan, Italy). Images were acquired using a HCX PL APO 63x/1.4 NA oil-immersion objective mounted on a Leica TCS SP8 confocal system (Leica Microsystems, Buccinasco, Milan, Italy) equipped with 4 excitation lasers. Image processing and quantification of total and nuclear GFP Mean Fluorescence Intensity (MFI) were performed using ImageJ v2.14 (Rasband, W.S., U.S. National Institutes of Health, Bethesda, MA, USA). To quantify PBX1 localization, a ratio of nuclear over total mean fluorescent intensity of the GFP signal (PBX1 expression) was calculated. A value of approximately 1 indicates that the protein localizes both in the nucleus and in the cytoplasm; a value below 1 indicates that the protein is predominantly in the cytoplasm.

### 2.10. Statistical Analyses

Statistical analyses were performed with GraphPad v9 (GraphPad Software Inc., La Jolla, CA, USA). Unpaired parametric *t*-test was used to determine statistical significance.

## 3. Results

### 3.1. Presentation of the Clinical Case

**Figure 1:**
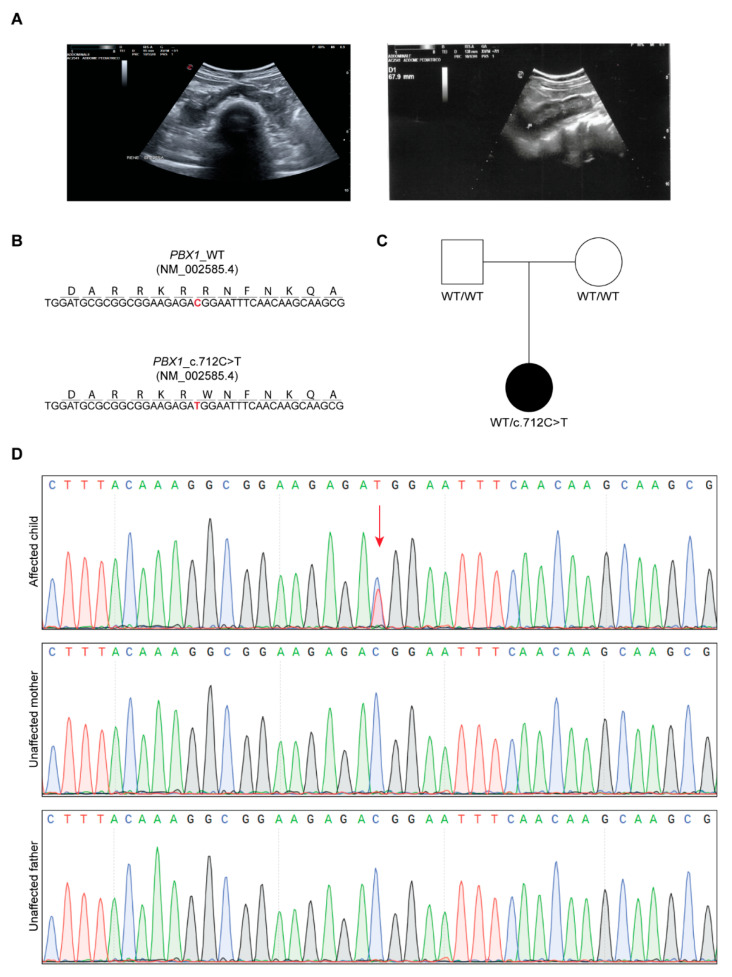
**Clinical and genetic features of the proband.** (**A**) Eco-scan of the proband’s abdomen showing the horseshoe kidney. (**B**) Nucleotide and amino acid sequence of part of exon 5 of WT and mutant *PBX1*. (**C**) Pedigree of the family. (**D**) Chromatograms of the proband (affected) and parents (unaffected); the missense change C>T in the proband is indicated with the red arrow.

The proband is a 4-year-old female (at the time of diagnosis) who presented with congenital hyperechoic horseshoe kidney (Figure 1A), hypotonia of the vocal cords, slight growth retardation and developmental delay. She was referred for genetic testing with a clinical suspicion of CAKUT. The child underwent surgical correction of the patent ductus arteriosus (PDA) five months after birth. When she was 2 years old, she suffered from recurrent acute pyelonephritis episodes that were treated with antibiotic prophylaxis. Moreover, she presented with a grade II bilateral vesicoureteral reflux (VUR) diagnosed at the age of two through a cystography.

### 3.2. Identification of a Novel PBX1 Missense Variant

**Figure 2:**
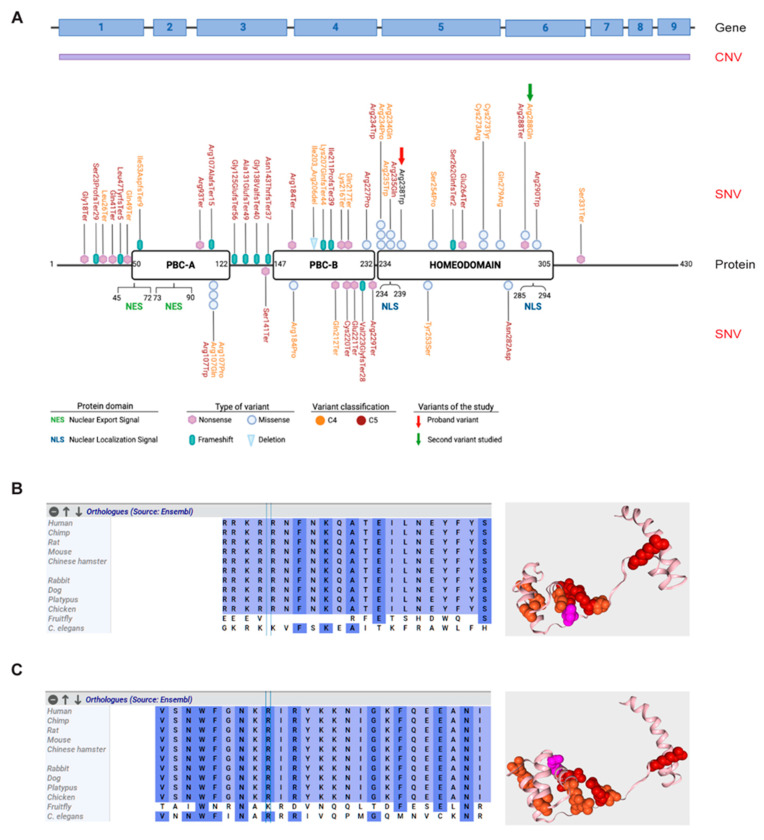
**PBX1 gene and protein organization and likely pathogenic and pathogenic variants within the gene.** (**A**) Schematic representation of *PBX1* gene (upper panel) and protein (lower panel). Copy number variants (CNVs) are indicated in purple as they usually encompass the whole gene. Likely pathogenic (C4—orange) and pathogenic (C5—red) single nucleotide variants (SNVs) are mapped on the functional domains of the protein: PBC-A, PBC-B and the homeodomain. Nuclear export signal (NES) and nuclear localization signal (NLS) are shown. The identified variant R238W is indicated with a red arrow, while the other variant studied in this work, R288Q, is indicated with a green arrow. (**B**,**C**) Phylogenetic aminoacidic conservation of the residue (left) and mapping of the studied variants within the protein structure [right; variant R238W (**B**) and variant R288Q (**C**)]. Likely pathogenic (C4) and pathogenic (C5) variants are shown in orange and red, respectively, while studied variants are shown in pink. Phylogenetic conservation was generated using Alamut software v1.12 (Sophia Genetics, Boston, MA, USA), while 3D protein viewer was generated using Varsome.

Based on clinical suspicion, CES was performed, focusing the tertiary analysis on an in silico list of genes associated with CAKUT (Table S1). The analysis led to the identification of a novel, never reported before, heterozygous variant located in exon 5 of *PBX1* [c.712C>T, NM_002585.4; Figure 1B]. The variant was then confirmed by Sanger sequencing and segregation studies unveiled its de novo nature, as both parents presented with a WT *PBX1* gene (Figure 1C,D). At the protein level, the variant maps in the C-terminal homeodomain, specifically in the first NLS, involved in PBX1 nuclear import from the cytoplasm (Figure 2A) [21,26] and results in the change of an arginine residue with a tryptophan at position 238 [(R238W); Figure 1B and Figure 2A]. The arginine residue at this position is phylogenetically highly conserved (10 over 12 species; Grantham distance: 101; Figure 2B) and its substitution is predicted to have a deleterious effect on protein function, according to PolyPhen2 and SIFT algorithms.

### 3.3. Genotype–Phenotype Association: A Snapshot of PBX1 Genetic Variants in the Literature

We initially performed a literature and genetic database review to obtain a list of all reported variants in the gene. As detailed in Table S2 and shown in Figure 2A, approximately 50 single-nucleotide variants (SNVs) have been reported to be involved in CAKUT or in inborn genetic diseases, as well as other congenital disorders, underlining the key role played by *PBX1* during development. Likely pathogenic (C4) and pathogenic (C5) SNVs are spread throughout the gene, with no mutational hotspots. It is however noteworthy that a significant amount of the identified variants involves the three conserved regions of the protein, including the protein–protein interacting PBC-A and PBC-B domains and the homeodomain. Most of them appear to be nonsense and frameshift variants, indicating a quantitative effect. Consistently, copy number variants (CNVs) involving *PBX1,* including whole [27] or partial gene deletions [16,28], and found in ≈20% of cases, may result in severe heterogenous phenotypes (Table 1).

### 3.4. Generation of an Ad Hoc Cellular Model to Study PBX1 Expression

**Figure 3:**
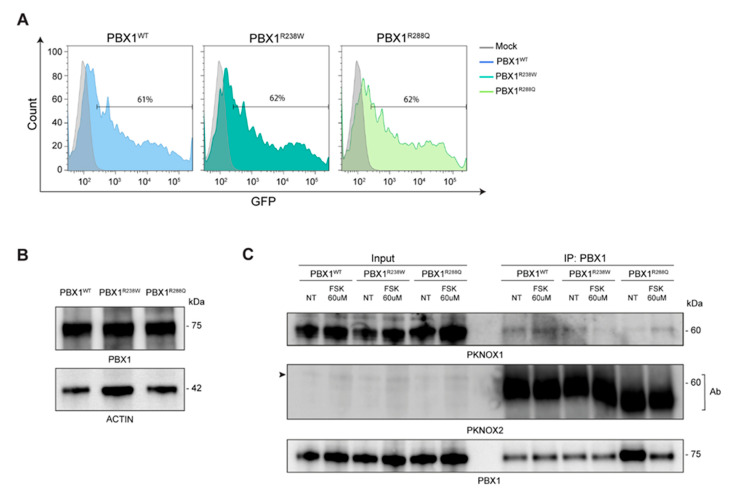
**PBX1 expression and interactions in basal and stimulated conditions**. (**A**) Cell transfection efficiency was evaluated by flow cytometry: non-GFP mock cells are used as negative control (grey line); data are shown as overlay of the different clones over mock cells. (**B**) Western blot analysis of total cell extracts of HEK293T cells transiently transfected with WT (PBX1^WT^) or mutant R238W (PBX1^R238Q^) and R288Q (PBX1^R288Q^) *PBX1*; molecular weights are shown on the right. Actin was used as a loading control. (**C**) Western blot analysis of immunoprecipitated (IP) WT or mutant R238W and R288Q *PBX1* in basal conditions and after FSK exposure. In the PKNOX2 blot, the arrow on the left indicates PKNOX2 band, while the bands in the immunoprecipitated lanes indicate the antibody (Ab) used for immunoprecipitation.

With the aim of providing the patient with a diagnosis, we set up a quick and easy experimental model to test the impact of the identified variant. A second mutation, [(c.863G>A, R288Q); Figure 2A], mapping in the second NLS, reported in the literature but never functionally validated, was used as control (Figure 2C) [32]. This dual experimental approach allowed us to compare the impact of the two variants and better characterize their role in modulating PBX1 activity. To this aim, a plasmid encoding WT *PBX1* was mutagenized to express the two variants. Mutagenesis was validated by Sanger sequencing. Subsequently, plasmids were transiently transfected into HEK293T cells that have very low levels of endogenous PBX1 expression. Transfection efficiency, evaluated by flow cytometry as GFP expression, was ≈60% and comparable in all experiments (Figure 3A). Western blot analysis was performed to determine the expression of WT, PBX1^R238W^ and PBX1^R288Q^. In line with the fact that the plasmid used for transfection encodes the predominant and longest PBX1 isoform, known as PBX1a, and that it is tagged with GFP, a band of ~75 kDa was detected, with no significant differences between WT and mutants (Figure 3B), indicating that the two variants did not affect protein translation or stability, as expected.

### 3.5. PBX1 Variants Did Not Alter Its Interaction with PKNOX1

**Figure 4:**
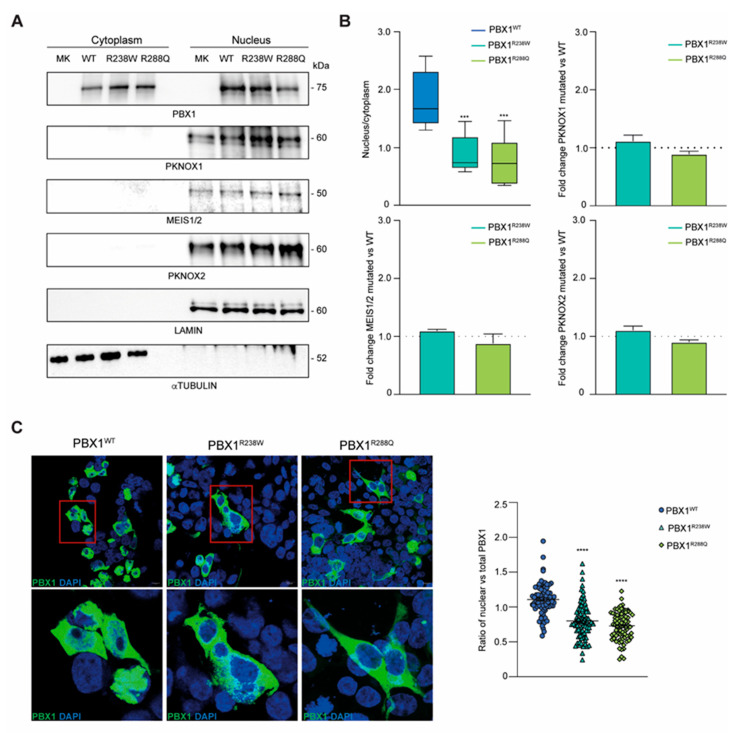
**PBX1 expression and intracellular localization of PBX1.** (**A**) PBX1 and its cofactors’ (PKNOX1, MEIS1/2 and PKNOX2) expression in cytoplasmic and nuclear fractions of HEK293T cells transiently transfected with WT or mutant R238W and R288Q *PBX1*. Lamin A/C and α-tubulin were used as nuclear and cytoplasmic fraction controls, respectively. (**B**) Densitometric quantification of nuclear PBX1 over the cytoplasmic counterpart (top left; n = 5). Ratio of PKNOX1 (top right), MEIS1/2 (bottom left) and PKNOX2 (bottom right) cofactors in PBX1 mutant clones (R238W and R288Q) over WT PBX1 (dotted line; n = 3). Cofactor protein bands were normalized on Lamin A/C. Error bars represent standard deviation (SD). Statistical analysis was performed using unpaired parametric *t*-test (mutant R238W and R288Q vs. WT; *p* < 0.001, ***). (**C**) Confocal microscopy images of WT and mutant *PBX1* (green signal, GFP). Nuclei were stained with DAPI (blue signal). Scale bar is 10 micrometer. Inset of PBX1 localization are shown in the lower panels. Quantification of nuclear vs. total GFP intensity was plotted. Ten images per clone were acquired and at least 20 cells per experiment were analyzed (n = 3). Error bars represent SEM. Statistical analysis was performed using unpaired parametric *t*-test (mutant R238W and R288Q vs. WT; *p* < 0.0001, ****).

We then checked whether PBX1 variants could impair interaction with its co-factors. To this purpose, we first induced a PKA-mediated phosphorylation of PBX1. Based on previously published data [33], PKA was activated by treating HEK293T-PBX1 cells with Forskolin (FSK; 60 microM; 2 h) and an anti-phospho-CREB (pCREB) antibody was used to confirm PKA activation [34]. We then investigated the interaction between PBX1 and its cofactors by performing an immunoprecipitation assay, both in basal and FSK-stimulated conditions. Results showed that, among all cofactors, PKNOX1 was the only one showing a weak interaction with PBX1^WT^, which did not significantly increase after FSK treatment (Figure 3C). The interaction between PKNOX1 and both mutant PBX1 appeared to be weaker than with PBX1^WT^ and was not further enhanced in FSK-activating conditions (Figure 3C). When blotting for PKNOX2, only the band corresponding to the antibody used to immunoprecipitate PBX1 appeared, suggesting that this cofactor may not directly or stably interact with PBX1. Moreover, no interactions between MEIS1/2 and PBX1 were highlighted, even though the expression of this cofactor was very low (Figure 4A), making it difficult to derive any conclusions.

### 3.6. R238W and R288Q Variants Impair Nuclear Localization of PBX1

Finally, since both variants mapped in the NLS of the protein, we determined whether they could influence nuclear translocation of PBX1. To this aim, cytoplasmic and nuclear fractions from HEK293T cells expressing PBX1^WT^, PBX1^R238W^ or PBX1^R288Q^ were isolated, and protein localization evaluated. Results showed a different distribution of PBX1^R238W^ between the two compartments compared to PBX1^WT^, with a more pronounced localization of the latter in the nucleus and a greater cytoplasmic retention of the mutant PBX1 (Figure 4A). PBX1^R288Q^, used as control, confirmed a defective translocation of the molecule into the nucleus, with a significantly lower amount present within this cellular compartment compared to the WT counterpart. Importantly, none of the variants completely prevented nuclear translocation, suggesting that a single intact NLS may be enough for partial translocation (Figure 4A,B). These results were further confirmed by confocal microscopy imaging. WT or mutant *PBX1* (both GFP-tagged) transfected HEK293T cells were stained with DAPI to identify the nucleus while looking at PBX1 localization. Images and their quantification confirmed that both PBX1^R238W^ and PBX1^R288Q^ clones were characterized by a reduced nuclear localization of the protein compared to WT counterpart, with a predominant expression within the cytoplasm (Figure 4C).

Lastly, we checked the expression levels of PBX1 interactors, including MEIS and PKNOX1/2. Data showed that all PBX1 co-factors localize within the nucleus with no significant differences in terms of expression, suggesting that the mutation effect is very specific on nuclear localization (Figure 4A,B).

Overall, the data obtained highlight the functional impact of the R238W and R288Q variants on PBX1 localization.

## 4. Discussion

CAKUT is an umbrella term that encompasses a wide range of kidney and urinary tract malformations, ranging from clinically silent to kidney failure phenotypes. Today, single-gene variants account for approximately 10–15% of CAKUT patients, while CNVs are detected in about 4–11% of patients [10,11,35]. Genotype–phenotype correlations are still a challenge considering genetic heterogeneity, variable expressivity and incomplete penetrance [36].

Chromosomal rearrangements involving *PBX1* have been identified and reported in pre-B-cell acute lymphoblastic leukemia development, in line with the role of this gene. More recently, genetic alterations affecting *PBX1* have been described as a monogenic cause of CAKUT, underlining the role of the encoded protein as a TF involved in kidney and urinary tract development. Loss-of-function variants, such as nonsense, splicing or frameshift variants are typically found in patients with renal hypoplasia and progressive kidney failure, similar to partial or whole gene deletions [28]. On the contrary, missense variants usually show milder phenotypes, including hyperechogenic or horseshoe kidneys, often without organ damage.

In this study, we functionally characterized a novel de novo missense heterozygous *PBX1* variant [c.712C>T, p.(Arg238Trp)] localized in the first NLS of the protein, identified in a 4-year-old proband with a mild phenotype to better understand its potential causal role in determining the clinical phenotype.

To this aim, we used a reproducible and easy-to-use cellular model to test the functional impact of the variant. Based on the results obtained, a few considerations can be inferred.

First, the proband variant PBX1^R238W^ significantly reduces (>50%) the nuclear localization of the protein. This result is in line with what was obtained when mimicking another variant, the PBX1^R288Q^, which maps in the second NLS of the protein and was reported as likely pathogenic in the literature. The obtained results underline the need to evaluate the mapping, at the protein level, of the identified variants to better assess their potential impact. Given the central role played by NLSs in correctly localizing the proteins, variants affecting these short nucleotide sequences have a deleterious impact. To investigate the consequences of the PBX1 genetic variant, we ectopically expressed both the WT and the mutated form of the gene in the HEK293T model. Since the identified variant was mapping in the NLS, we were primarily interested in comparatively assessing the sub-cellular localization of the resulting protein. For this reason, despite being a tumor-derived and not a kidney-specific cell line, HEK293T cells represented a suitable model for our purposes.

Second, considering the reduced nuclear localization of the PBX1^R238W^ and the fact that it is a TF, we investigated whether the transcription of some of its potential targets could be impaired or reduced because of the variant. Several targets have been reported in the literature [19], although most of them have been described only in the leukemic context. Results obtained in this work, investigating some of these known targets (*STAT3*, *PAX6*, *JUN e HIF1alpha*) showed no significant reduction in our PBX1 models. By looking at these results, one possibility is that the investigated targets may be tumor-specific while not playing any role in the renal phenotype. Surfing in the literature, there is still a lack of evidence on which could be the actual PBX1 targets involved in embryogenesis and kidney development. In line with this consideration, transcriptomics approaches may represent a useful tool to identify them and possibly clarify the main players involved. However, even though of scientific and clinical interest, this type of investigation was out of the scope of this study, which only aimed at determining the impact of the identified variant in causing patient’s phenotype.

Third, segregation studies and functional characterization of genetic variants allow for a better understanding of their pathogenetic role. In the case of the studied variant, family segregation studies that excluded the presence of the variant in proband’s parents, both healthy subjects, and the reduced localization within the nucleus allowed the study to highlight the pathogenetic role of the variant, elucidating the clinical phenotype observed in the patient. Indeed, the impact of PBX1^R238W^, together with the fact that its role can be critical during the development, may explain the involvement in renal phenotypes. These results underline the importance of combining clinics with laboratory work to shed lights on the genetic cause of complex disease phenotypes.

Overall, the findings of this work emphasize the importance of determining the genetic basis of a clinical condition by using the correct genetic tests and elucidating the functional consequences of the identified variants, if still uncertain, to better understand the underlying pathogenetic mechanisms. This knowledge is essential for providing correct counseling, guiding clinical management of affected individuals and, if available, optimizing therapeutic strategies.

## Figures and Tables

**Table 1:** ***PBX1* genetic variants reported in the literature and in the main genetic variant databases. CHR:** chromosome; **ACMG:** American College of Medical Genetics and Genomics; **C5:** pathogenic variant; **C4:** likely pathogenic variant; **C3:** variant of unknown significance—VUS; **WGD**: whole gene deletion; **WG**: whole gene; **CAKUTHED:** Congenital Anomalies of Kidney and Urinary Tract Syndrome with or without Hearing Loss, Abnormal Ears, or Developmental Delay; **ID**: intellectual disability and pleiotropic developmental defects; **CA**: congenital anomalies. **REF:** references; **S:** submitted in genetic databases (ClinVar); **NA:** not available. Variants are mapped on hg19. The Table is updated as of 13 December 2024.

CHR	Type of Variant	ACMG	REF	Phenotype
1: 149825831-180236332	Copy number gain (WGD)	Clinvar (C5); Franklin (C5)	[29]	ID; CA
1: 157717036-175990383	Copy number gain (WGD)	Clinvar (C5); Franklin (C5)	[30]	ID
1: 159449677-166864323	Copy number loss (WGD)	Clinvar (C5); Franklin (C5)	[30]	ID
1: 161710697-173934292	Copy number loss (WGD)	Clinvar (C5); Franklin (C5)	[30]	ID
1: 162009840-167449900	Copy number loss (WGD)	Clinvar (C5); Franklin (C5)	[30]	ID
1: 163352313-175846158	Copy number loss (WGD)	Clinvar (C5); Franklin (C5)	[29]	ID; CA
1: 164516783-165658640	Copy number loss (WGD)	Clinvar (C3); Franklin (C5)	[30]	ID
1: 163815650-164647742	Copy number gain (partial gene deletion, exon 1–2)	Clinvar (C3); Franklin (C5)	S	NA
1: 164571371-175708060	Copy number loss (partial gene deletion, exon 3–9)	Clinvar (C5); Franklin (C5)	S	NA
1: 161924068-164761399	Copy number loss (partial gene deletion, exon 1–2)	Clinvar (C4); Franklin (C5)	S	NA
1: 164749028-165296253	Copy number gain (partial gene deletion, exon 3–9)	Clinvar (C3); Franklin (C4)	S	NA
1: 162330810-171532331	Copy number loss (WGD)	Clinvar (C5); Franklin (C5)	S	NA
1: 160417296-166197042	Copy number loss (WGD)	Clinvar (C5); Franklin (C5)	S	NA
1: 130980840-248900000	Duplication (WG)	Clinvar (C3); Franklin (C5)	S	Paragangliomas; gastrointestinal stromal tumor; parathyroid carcinoma
1: 164608682-169216098	Copy number loss (partial gene deletion, exon 3–9)	Clinvar (C4); Franklin (C5)	NA	NA
1: 163093021-168991239	Copy number loss (WGD)	Clinvar (C5); Franklin (C5)	NA	NA
1: 157321299-167391423	Copy number loss (WGD)	Clinvar (C5); Franklin (C5)	NA	NA
1: 164690187-164835917	Copy number gain (partial gene deletion, exon 3–9)	Clinvar (C3); Franklin (C4)	NA	NA
1: 160369890-175796325	Copy number loss (WGD)	Clinvar (C5); Franklin (C5)	[31]	Growth retardation, microcephaly, intellectual disability, dysmorphism
1: 849467-249224684	Copy number gain (WGD)	Clinvar (C5); Franklin (C5)	[29]	ID; CA
1: 849467-249224684	Copy number gain (WGD)	Clinvar (C5); Franklin (C5)	[29]	ID; CA
1: 161676893-184071723	Copy number gain (WGD)	Clinvar (C5); Franklin (C5)	[29]	ID; CA
1: 163193466-166058476	Deletion (WG)	Franklin (C5)	[16]	8 patients showing different phenotypes: face and head anomalies, renal and urinary anomalies, genitalia anomalies, gonadal/uterine anomalies, skeletal anomalies, cardiac anomalies, hearing loss, pulmonary hypoplasia
1: 164749027-164786500	Copy number loss (partial gene deletion, exon 3–6)	Franklin (C5)	[28]	
1q23.3q24.1 1q23.3q24.3	2.46 Mb deletion (WG) 6.2 Mb deletion (WG)	C5	[27]	Deafness, ID, face and head anomalies, renal and urinary anomalies

## Data Availability

For data inquiries, please contact Silvia Deaglio (silvia.deaglio@unito.it).

## Supplementary Materials

The following supporting information can be downloaded at https://www.mdpi.com/article/10.3390/genes16111346/s1. Supplementary materials contain: Table S1. List of genes associated with CAKUT phenotype and Table S2. PBX1 SNV genetic variants reported in literature and in the main genetic variant databases.

## Abbreviations

ACMG	American College of Medical Genetics and Genomics
C3-VUS	variant of unknown significance
CAKUT	Congenital Anomalies of the Kidney and Urinary Tract
CES	Clinical exome sequencing
CKD	chronic kidney disease
CNVs	copy number variants
FSK	forskolin
IP	immunoprecipitated
MEIS	Myeloid Ecotropic Integration Site
NES	nuclear export signal
NLS	nuclear localization signal
*PBX1*	Pre-B cell Leukemia Factor 1
pCREB	phospho-CREB
PDA	patent ductus arteriosus
PKNOX1	PBX-regulating protein 1
PKNOX2	PBX-regulating protein 2
SNVs	single-nucleotide variants
TALE	three-amino-acid loop extension
TF	transcription factor
VUR	vesicoureteral reflux
WB	Western blot
WT	wild-type
