# Supplementary material for: Functional Characterization of a Novel PBX1 De Novo Missense Variant Identified in a Pediatric Patient with CAKUT
Source: Genes (Basel). 2025 Nov 7;16(11):1346. doi: 10.3390/genes16111346 (PMC12652685; doi:10.3390/genes16111346)
Supplement: Supplementary file 1 [file genes-16-01346-s001.zip › genes-3940097-supplementary.pdf]

Supplementary tables

Table S1. List of genes associated with CAKUT phenotype.		
**Gene**	**Coding protein**	**Reference Sequence**
ACE	Angiotensin I-Converting Enzyme	NM_000789.4
ACTB	Beta-Actin	NM_001101.5
ACTG1	Gamma-Actin	NM_001614.5
ACTG2	Gamma 2-Actin, Smooth Muscle	NM_001615.4
AFF3	ALF Transcription Elongation Factor 3	NM_001386135.1
AGT	Angiotensinogen	NM_001384479.1
AGTR1	Angiotensin II Receptor, Type 1	NM_000685.5
ALMS1	ALMS1 Centrosome and Basal Body Associated Protein	NM_001378454.1
AMER1	APC Membrane Recruitment Protein 1	NM_152424.4
ANOS1	Anosmin 1	NM_000216.4
ATP7A	ATPase Copper Transporting Alpha	NM_000052.7
BCOR	BCL6 Corepressor	NM_001123385.2
BICC1	BicC Family RNA-Binding Protein 1	NM_001080512.3
BMP4	Bone Morphogenetic Protein 4	NM_001202.6
BNC2	Basonuclin-2	NM_017637.6
BSND	Barttin CLCNK-Type Accessory Subunit Beta	NM_057176.3
CCBE1	Collagen And Calcium-Binding EGF Domain-Containing Protein 1	NM_133459.4
CCNQ	Cyclin Q	NM_152274.5
CD151	CD151 Antigen	NM_004357.5
CDKN1C	Cyclin-Dependent Kinase Inhibitor 1C	NM_001122630.2
CDX2	Caudal-Type Homeobox Transcription Factor 2	NM_001265.6
CHD1L	Chromodomain Helicase DNA-Binding Protein 1-Like	NM_004284.6
CHD7	Chromodomain Helicase DNA-Binding Protein 7	NM_017780.4
CHRM3	Cholinergic Receptor, Muscarinic 3	NM_001375978.1
CHRNA3	Cholinergic Receptor, Neuronal Nicotinic, Alpha Polypeptide 3	NM_000743.5
CHST14	Carbohydrate Sulfotransferase 14	NM_130468.4
CISD2	CDGSH Iron Sulfur Domain Protein 2	NM_001008388.5
DCHS1	Dachsous Cadherin-Related 1	NM_003737.4
DHCR7	7-Dehydrocholesterol Reductase	NM_001360.3

DSTYK	Dual Serine/Threonine And Tyrosine Protein Kinase	NM_015375.3
DYRK1A	Dual-Specificity Tyrosine Phosphorylation Regulated Kinase 1A	NM_001347721.2
ESCO2	Establishment Of Sister Chromatid Cohesion N-Acetyltransferase 2	NM_001017420.3
ETFA	Electron Transfer Flavoprotein, Alpha Polypeptide	NM_000126.4
ETFB	Electron Transfer Flavoprotein, Beta Polypeptide	NM_001985.3
ETFDH	Electron Transfer Flavoprotein Dehydrogenase	NM_004453.4
EYA1	EYA Transcriptional Coactivator And Phosphatase 1	NM_000503.6
FANCA	Fanconi Anemia Complementation Group A	NM_000135.4
FANCB	Fanconi Anemia Complementation Group B	NM_001018113.3
FANCC	Fanconi Anemia Complementation Group C	NM_000136.3
FANCD2	Fanconi Anemia Complementation Group D2	NM_001018115.3
FANCE	Fanconi Anemia Complementation Group E	NM_021922.3
FANCI	Fanconi Anemia Complementation Group I	NM_001113378.2
FANCL	Fanconi Anemia Complementation Group L	NM_018062.4
FAT4	FAT Atypical Cadherin 4	NM_001291303.3
FGF10	Fibroblast Growth Factor 10	NM_004465.2
FGF20	Fibroblast Growth Factor 20	NM_019851.3
FGFR2	Fibroblast Growth Factor Receptor 2	NM_000141.5
FLNA	Filamin A	NM_001110556.2
FOXC1	Forkhead Box C1	NM_001453.3
FOXP1	Forkhead Box P1	NM_001349338.3
FRAS1	Fraser Extracellular Matrix Complex Subunit 1	NM_025074.7
FREM1	FRAS1-Related Extracellular Matrix Protein 1	NM_001379081.2
FREM2	FRAS1-Related Extracellular Matrix Protein 2	NM_207361.6
GATA3	GATA-Binding Protein 3	NM_001002295.2
GDF6	Growth Differentiation Factor 6	NM_001001557.4
GLI3	GLI Family Zinc Finger 3	NM_000168.6
GPC3	Glypican 3	NM_004484.4
GRIP1	Glutamate Receptor Interacting Protein 1	NM_001366722.1
HNF1B	HNF1 homeobox B	NM_000458.4
HOXA13	Homeobox A13	NM_000522.5
HPSE2	Heparanase 2	NM_021828.5

HSPA9	Heat Shock Protein Family A (Hsp70) Member 9	NM_004134.7
HYLS1	HYLS1 Centriolar And Ciliogenesis-Associated Protein	NM_001134793.2
ITGA3	Integrin Subunit Alpha 3	NM_002204.4
ITGA8	Integrin Subunit Alpha 8	NM_003638.3
JAG1	Jagged 1	NM_000214.3
JAM3	Junctional Adhesion Molecule 3	NM_032801.5
KAT6B	Lysine Acetyltransferase 6B	NM_012330.4
KCTD1	Potassium Channel Tetramerization Domain-Containing Protein 1	NM_001142730.3
KDM6A	Lysine Demethylase 6A	NM_001291415.2
KIF14	Kinesin Family Member 14	NM_014875.3
KMT2D	Lysine-Specific Methyltransferase 2D	NM_003482.4
KRAS	KRAS Proto-Oncogene, GTPase	NM_004985.5
KYNU	Kynureninase	NM_003937.3
LIFR	Leukemia Inhibitory Factor Receptor	NM_001127671.2
LMNA	Lamin A/C	NM_170707.4
LRIG2	Leucine-Rich Repeats- and Immunoglobulin-Like Domains-Containing Protein 2	NM_014813.3
LRP4	Low Density Lipoprotein Receptor-Related Protein 4	NM_002334.4
MUC1	Mucin 1, Cell Surface Associated	NM_001204286.1
MYOCD	Myocardin	NM_001146312.3
NEK8	NIMA-Related Kinase 8	NM_178170.3
NFIA	Nuclear Factor I A	NM_001134673.4
NIPBL	NIPBL Cohesin Loading Factor	NM_133433.4
NOTCH2	Notch Receptor 2	NM_024408.4
NPHP3	Nephrocystin 3	NM_153240.5
NRIP1	Nuclear Receptor-Interacting Protein 1	NM_003489.4
NSDHL	NAD(P)H Steroid Dehydrogenase-Like Protein	NM_015922.3
OCRL	Inositol Polyphosphate-5-Phosphatase	NM_000276.4
OFD1	OFD1 Centriole And Centriolar Satellite Protein	NM_003611.3
PAX2	Paired Box Gene 2	NM_000278.5
PBX1	Pre-B-Cell Leukemia Transcription Factor 1	NM_002585.4
PEX1	Peroxisomal Biogenesis Factor 1	NM_000466.3
PEX5	Peroxisomal Biogenesis Factor 5	NM_001351132.2

PIGL	Phosphatidylinositol Glycan Anchor Biosynthesis Class L	NM_004278.4
PIGN	Phosphatidylinositol Glycan Anchor Biosynthesis Class N	NM_176787.5
PIGO	Phosphatidylinositol Glycan Anchor Biosynthesis Class O	NM_032634.4
PIGV	Phosphatidylinositol Glycan Anchor Biosynthesis Class V	NM_017837.4
PMM2	Phosphomannomutase 2	NM_000303.3
PORCN	Porcupine O-Acyltransferase	NM_203475.3
PROK2	Prokineticin 2	NM_001126128.2
PTPN11	Protein-Tyrosine Phosphatase, Non-Receptor Type 11	NM_002834.5
RAD51C	RAD51 Paralog C	NM_058216.3
RAI1	Retinoic Acid-Induced Gene 1	NM_030665.4
RECQL4	RecQ Protein-Like 4	NM_004260.4
REN	Renin	NM_000537.4
RET	Ret proto-oncogene	NM_020975.6
ROBO1	Roundabout Guidance Receptor 1	NM_002941.4
ROBO2	Roundabout Guidance Receptor 2	NM_001395656.1
ROR2	Receptor Tyrosine Kinase-Like Orphan Receptor 2	NM_004560.4
RPGRIP1L	RPGRIP1 Like	NM_015272.5
SALL1	Spalt-Like Transcription Factor 1	NM_002968.3
SALL4	Spalt-Like Transcription Factor 4	NM_020436.5
SEMA3E	Semaphorin 3E	NM_012431.3
SETBP1	SET-Binding Protein 1	NM_015559.3
SF3B4	Splicing Factor 3B, Subunit 4	NM_005850.5
SHH	Sonic Hedgehog Signaling Molecule	NM_000193.4
SIX1	SIX Homeobox 1	NM_005982.4
SIX5	SIX Homeobox 5	NM_175875.5
SLX4	SLX4 Structure-Specific Endonuclease Subunit	NM_032444.4
SOX11	SRY-Box Transcription Factor 11	NM_003108.4
SOX17	SRY-Box Transcription Factor 17	NM_022454.4
SOX9	SRY-Box Transcription Factor 9	NM_000346.4
SRCAP	SNF2-Related CBP Activator Protein	NM_006662.3
STRA6	Signaling Receptor and Transporter Of Retinol	NM_022369.4
TBC1D1	TBC1 Domain Family, Member 1	NM_001396959.1

TBX18	T-Box Transcription Factor 18	NM_001080508.3
TFAP2A	Transcription Factor AP2-Alpha	NM_001372066.1
TMCO1	Transmembrane and Coiled-Coil Domains Protein 1	NM_016292.3
TNXB	Tenascin XB	NM_001365276.2
TP63	Tumor Protein p63	NM_003722.5
TRAP1	Tumor Necrosis Factor Receptor-Associated Protein 1	NM_016292.3
UBR1	Ubiquitin-Protein Ligase E3 Component n-Recognin 1	NM_174916.3
UMOD	Uromodulin	NM_003361.4
UPK3A	Uroplakin 3A	NM_006953.4
DYNC2I1	WD Repeat Domain 60 or Dynein 2 Intermediate Chain 1	NM_018051.5
WFS1	Wolframin ER Transmembrane Glycoprotein	NM_006005.3
WNT3	Wnt Family Member 3	NM_030753.5
WNT4	Wnt Family Member 4	NM_030761.5
WNT5A	Wnt Family Member 5A	NM_003392.7
XRCC2	X-Ray Repair Cross Complementing 2	NM_005431.2
ZIC3	Zic Family, Member 3	NM_003413.4
ZMPSTE24	Zinc Metallopeptidase STE24	NM_005857.5
ZMYM2	Zinc Finger, MYM-Type Containing 2	NM_197968.4

Table S2. ***PBX1*** **SNV genetic variants reported in literature and in the main genetic variant databases.**
CHR: chromosome; **ACMG:** American College of Medical Genetics and Genomics; **C5:** Pathogenic variant; **C4:** likely pathogenic variant; **C3:** variant of unknown significance – VUS; **Int:** intron; **CAKUTHED:** Congenital Anomalies of Kidney and Urinary Tract Syndrome with or without Hearing Loss, Abnormal Ears, or Developmental Delay; **IGD:** inborn genetic disease; **ID**: intellectual disability and pleiotropic developmental defects; **CHD:** congenital heart disease; **CDH**: congenital diaphragmatic hernia; **MOM:** multiple organ malformations; **REF:** references; **S:** submitted in genetic databases; **P**: published; **NA:** not available.
Variants refer to the canonical transcript NM_002585.4. The Table is updated as of July 8, 2024**.**

CHR 1	EXON/ INTRON	CODING SEQUENCE	PROTEIN	TYPE OF VARIANT	ACMG	REF	PHENOTYPE
164529077	1	c.22_37dup	p.(Gly13AspfsTer53)	Frameshift	C3	S	NA
164529091	1	c.32A>G	p.(His11Arg)	Missense	C3	S	NA
164529111	1	c.52G>T	p.(Gly18Ter)	Nonsense	C5	S	NA
164529126	1	c.67del	p.(Ser23ProfsTer29)	Frameshift	C5	S	CAKUTHED
164529136	1	c.77T>A	p.(Leu26Ter)	Nonsense	C4	S	NA
164529153	1	c.94G>A	p.(Gly32Arg)	Missense	C3	S	NA
164529172	1	c.113G>A	p.(Gly38Glu)	Missense	C3	S	IGD
164529180	1	c.121C>T	p.(Gln41Ter)	Nonsense	C5	S	CAKUTHED
164529196	1	c.140del	p.(Leu47TyrfsTer5)	Frameshift	C5	[1]	ID
164529204	1	c.145C>T	p.(Gln49Ter)	Nonsense	C4	S	CAKUTHED
164529211	1	c.152_155dup	p.(Ile53AspfsTer9)	Frameshift	C4	S	CAKUTHED
164532547	Int. 2	c.265+2_265+5del	NA	Splicing	C5	S	CAKUTHED
164532549	Int. 2	c.265+1G>A	NA	Splicing	C4	S	CAKUTHED
164761736	3	c.271A>C	p.(Ser91Arg)	Missense	C3	S	NA
164761742	3	c.277C>T	p.(Arg93Ter)	Nonsense	C4/ C5	S	CAKUTHED
164761779	3	c.314T>A	p.(Leu105Gln)	Missense	C3	S	NA
164761783	3	c.318dup	p.(Arg107AlafsTer15)	Frameshift	C5	S	CAKUTHED
164761784	3	c.319C>T	p.(Arg107Trp)	Missense	C4/ C5	[2]	CAKUTHED
164761785	3	c.320G>A	p.(Arg107Gln)	Missense	C4	S	CAKUTHED
164761785	3	c.320G>C	p.(Arg107Pro)	Missense	C4	S	CAKUTHED
164761796	3	c.331A>G	p.(Met111Val)	Missense	C3	S	CAKUTHED
164761797	3	c.332T>A	p.(Met111Lys)	Missense	C3	S	CAKUTHED
164761814	3	c.349G>C	p.(Val117Leu)	Missense	C3	S	NA
164761835	3	c.370_371dup	p.(Gly125GlufsTer56)	Frameshift	C5	S	NA
164761842	3	c.393_401dup	p.(Ala133_Ala135dup)	Inframe indel	C3	S	NA

164761857	3	c.392del	p.(Ala131GlufsTer49)	Frameshift	C5	S	NA
164761859	3	c.402_404dup	p.Ala135dup	Inframe indel	C3	S	NA
164761878	3	c.413_419del	p.(Gly138ValfsTer40)	Frameshift	C5	[3]	CAKUTHED
164761878	3	c.413G>T	p.(Gly138Val)	Missense	C3	S	CAKUTHED
164761887	3	c.422C>G	p.(Ser141Ter)	Nonsense	C5	S	NA
164761893	3	c.428del	p.(Asn143ThrfsTer37)	Frameshift	C5	[4]	CAKUTHED
164761968	3	c.503A>T	p.(Tyr168Phe)	Missense	C3	S	NA
164768934	Int. 3	c.511-2A>G	NA	Splicing	C5	[4]	CAKUTHED
164768967	4	c.542A>G	p.(Asn181Ser)	Missense	C3	S	NA
164768970	4	c.545T>C	p.(Leu182Pro)	Missense	C3	S	NA
164768975	4	c.550C>T	p.(Arg184Ter)	Nonsense	C5	[1, 3, 4]	CAKUTHED
164768976	4	c.551G>C	p.(Arg184Pro)	Missense	C4	[1, 5]	CAKUTHED; CHD
164769030	4	c.608_619del	p.(Ile203_Arg206del)	Inframe indel	C4	S	CAKUTHED
164769040	4	c.616del	p.(Arg206AlafsTer18)	Frameshift	C3	S	NA
164769043	4	c.618dup	p.(Lys207GlnfsTer44)	Frameshift	C4	S	CAKUTHED
164769052	4	c.627C>A	p.(Ser209Arg)	Missense	C3	S	NA
164769055	4	c.630_631del	p.(Ile211ProfsTer39)	Frameshift	C5	S	CAKUTHED
164769059	4	c.634C>T	p.(Gln212Ter)	Nonsense	C4	S	CAKUTHED
164769071	4	c.646A>T	p.(Lys216Ter)	Nonsense	C4	S	CAKUTHED
164769074	4	c.649C>T	p.(Gln217Ter)	Nonsense	C4	S	NA
164769085	4	c.660C>A	p.(Cys220Ter)	Nonsense	C5	S	NA
164769086	4	c.661G>T	p.(Glu221Ter)	Nonsense	C5	[6]	CAKUTHED
164769091	4	c.667dup	p.(Val223GlyfsTer28)	Frameshift	C5	S	CAKUTHED
164769105	4	c.680G>C	p.(Arg227Pro)	Missense	C5	[1]	ID
164769110	4	c.685C>T	p.(Arg229Ter)	Nonsense	C5	[4]	CAKUTHED
164769125	4	c.700C>T	p.(Arg234Trp)	Missense	C5	[7]	CDH
164769126	4	c.701G>C	p.(Arg234Pro)	Missense	C4	[1]	ID
164769126	4	c.701G>A	p.(Arg234Gln)	Missense	C4	S	CAKUTHED
164769128	Int. 4	c.701+2T>G	NA	Splicing	C4	S	CAKUTHED

164776780	5	c.703C>T	p.(Arg235Trp)	Missense	C4	S	CAKUTHED
164776781	5	c.704G>A	p.(Arg235Gln)	Missense	C5	[1, 8]	ID
164776835	5	c.758A>C	p.(Tyr253Ser)	Missense	C4	S	CAKUTHED
164776837	5	c.760T>C	p.(ser254Pro)	Missense	C4	[1]	ID
164776860	5	c.783dup	p.(Ser262GlnfsTer2)	Frameshift	C5	[1]	ID
164776867	5	c.790G>T	p.(Glu264Ter)	Nonsense	C5	[1, 3, 4]	ID; CAKUTHED
164776878	5	c.801G>C	p.(Glu267Asp)	Missense	C3	S	NA
164776894	5	c.817T>C	p.(Cys273Arg)	Missense	C4	S	CAKUTHED
164776895	5	c.818G>A	p.(Cys273Tyr)	Missense	C4	S	CAKUTHED
164776913	5	c.836A>G	p.(Gln279Arg)	Missense	C4	[9]	CAKUTHED
164781226	Int. 5	c.838-1G>C	NA	Splicing	C4	S	NA
164781233	6	c.844A>G	p.(Asn282Asp)	Missense	C5	S	NA
164781251	6	c.862C>T	p.(Arg288Ter)	Nonsense	C5	[1, 10]	ID; CAKUTHED
164781252	6	c.863G>A	p.(Arg288Gln)	Missense	C4	[11]	NA
164781254	6	c.865A>C	p.(Ile289Leu)	Missense	C3	S	CAKUTHED
164781257	6	c.868C>T	p.(Arg290Trp)	Missense	C5	[12, 13]	MOM
164781280	6	c.891A>C	p.(Lys297Asn)	Missense	C3	S	NA
164781287	6	c.898G>A	p.(Glu300Lys)	Missense	C3	S	NA
164781375	6	c.986C>A	p.(Pro329His)	Missense	C3	S	IGD
164781381	6	c.992C>A	p.(Ser331Ter)	Nonsense	C4	[14]	CAKUTHED
164789357	7	c.1046G>A	p.(Ser349Asn)	Missense	C3	S	IGD
164789359	7	c.1048G>A	p.(Val350Met)	Missense	C3	S	NA
164789410	7	c.1099G>A	p.(Val367Met)	Missense	C3	S	NA
164790786	8	c.1123C>T	p.(Arg375Cys)	Missense	C3	S	NA
164790838	8	c.1175A>G	p.(Gln392Arg)	Missense	C3	S	NA
164815824	9	c.1204A>C	p.(Asn402His)	Missense	C3	S	IGD
164815866	9	c.1246T>A	p.(Ser416Thr)	Missense	C3	S	IGD
164815885	9	c.1265G>A	p.(Gly422Asp)	Missense	C3	S	NA

References

1. Slavotinek, A., et al., *De novo, deleterious sequence variants that alter the transcriptional activity of the homeoprotein PBX1 are associated with intellectual disability and pleiotropic developmental defects.* Hum Mol Genet, 2017. **26**(24): p. 4849-4860.
2. Arts, P., et al., *Paternal mosaicism for a novel PBX1 mutation associated with recurrent perinatal death: Phenotypic expansion of the PBX1-related syndrome.* Am J Med Genet A, 2020. **182**(5): p. 1273-1277.
3. Riedhammer, K.M., et al., *Identification of a Novel Heterozygous De Novo 7-bp Frameshift Deletion in PBX1 by Whole-Exome Sequencing Causing a Multi-Organ Syndrome Including Bilateral Dysplastic Kidneys and Hypoplastic Clavicles.* Front Pediatr, 2017. **5**: p. 251.
4. Heidet, L., et al., *Targeted Exome Sequencing Identifies PBX1 as Involved in Monogenic Congenital Anomalies of the Kidney and Urinary Tract.* J Am Soc Nephrol, 2017. **28**(10): p. 2901-2914.
5. Alankarage, D., et al., *Functional characterization of a novel PBX1 de novo missense variant identified in a patient with syndromic congenital heart disease.* Hum Mol Genet, 2020. **29**(7): p. 1068-1082.
6. Stippel, M., et al., *Renal and Skeletal Anomalies in a Cohort of Individuals With Clinically Presumed Hereditary Nephropathy Analyzed by Molecular Genetic Testing.* Front Genet, 2021. **12**: p. 642849.
7. Kammoun, M., et al., *Genetic profile of isolated congenital diaphragmatic hernia revealed by targeted next-generation sequencing.* Prenat Diagn, 2018. **38**(9): p. 654-663.
8. Eozenou, C., et al., *The TALE homeodomain of PBX1 is involved in human primary testis-determination.* Hum Mutat, 2019. **40**(8): p. 1071-1076.

9. Bertoli-Avella, A.M., et al., *Successful application of genome sequencing in a diagnostic setting: 1007 index cases from a clinically heterogeneous cohort.* Eur J Hum Genet, 2021. **29**(1): p. 141-153.

10. Nie, L., et al., *A Pathogenic Variant of PBX1 Identified by Whole Exome Sequencing in a Chinese CAKUTHED Case.* Nephron, 2023. **147**(5): p. 311-316.

11. Blasi, F., et al., *A tale of TALE, PREP1, PBX1, and MEIS1: Interconnections and competition in cancer.* Bioessays, 2017. **39**(5).

12. Mathiasen, L., et al., *The flexibility of a homeodomain transcription factor heterodimer and its allosteric regulation by DNA binding.* FEBS J, 2016. **283**(16): p. 3134-54.

13. Ruscitti, F., et al., *An example of parenchymal renal sparing in the context of complex malformations due to a novel mutation in the PBX1 gene.* Birth Defects Res, 2022. **114**(12): p. 674-681.

14. Petzold, F., et al., *Novel somatic PBX1 mosaicism likely masking syndromic CAKUT in an adult with bilateral kidney hypoplasia.* Clin Kidney J, 2022. **15**(7): p. 1333-1339.
